# Cannabinoids disrupt memory encoding by functionally isolating hippocampal CA1 from CA3

**DOI:** 10.1371/journal.pcbi.1005624

**Published:** 2017-07-07

**Authors:** Roman A. Sandler, Dustin Fetterhoff, Robert E. Hampson, Sam A. Deadwyler, Vasilis Z. Marmarelis

**Affiliations:** 1 Department of Biomedical Engineering, University of Southern California, Los Angeles, California, United States of America; 2 Department of Physiology & Pharmacology, Wake Forest University, Winston-Salem, North Carolina, United States of America; University College London, UNITED KINGDOM

## Abstract

Much of the research on cannabinoids (CBs) has focused on their effects at the molecular and synaptic level. However, the effects of CBs on the dynamics of neural circuits remains poorly understood. This study aims to disentangle the effects of CBs on the functional dynamics of the hippocampal Schaffer collateral synapse by using data-driven nonparametric modeling. Multi-unit activity was recorded from rats doing an working memory task in control sessions and under the influence of exogenously administered tetrahydrocannabinol (THC), the primary CB found in marijuana. It was found that THC left firing rate unaltered and only slightly reduced theta oscillations. Multivariate autoregressive models, estimated from spontaneous spiking activity, were then used to describe the dynamical transformation from CA3 to CA1. They revealed that THC served to functionally isolate CA1 from CA3 by reducing feedforward excitation and theta information flow. The functional isolation was compensated by increased feedback excitation within CA1, thus leading to unaltered firing rates. Finally, both of these effects were shown to be correlated with memory impairments in the working memory task. By elucidating the circuit mechanisms of CBs, these results help close the gap in knowledge between the cellular and behavioral effects of CBs.

## Author summary

Research into cannabinoids (CBs) over the last several decades has found that they induce a large variety of oftentimes opposing effects on various neuronal receptors and processes. Due to this plethora of effects, disentangling how CBs influence neuronal circuits has proven challenging. This paper contributes to our understanding of the circuit level effects of CBs by using data driven modeling to examine how THC affects the input-output relationship in the Schaffer collateral synapse in the hippocampus. It was found that THC functionally isolated CA1 from CA3 by reducing feedforward excitation and theta information flow while simultaneously increasing feedback excitation within CA1. By elucidating the circuit mechanisms of CBs, these results help close the gap in knowledge between the cellular and behavioral effects of CBs.

## Introduction

Recent years have seen a resurgence of interest in the therapeutic role of cannabinoids (CBs) for several diseases and neurophyschiatric disorders such as psychosis, anxiety disorders, PTSD, and multiple sclerosis [1, 2]. In particular, CB agonists have shown promising but mixed results in the treatment of epilepsy, as various types of agonists at various doses have been shown to be both pro- and anticonvulsant [3–8]. Parallel to increasing therapeutic research, much work has been done on the chemical structure of various cannabinoids and cannabinoid receptors, along with their cellular interactions and pharmacology [9].

Nonetheless, between the large bodies of literature on cannabinoids from chemical, disease, and behavioral perspectives, much less work has been done to explore the effects of cannabinoids on the neural circuit level. This is particularly important since a wide range of complex and often opposing effects have been attributed to cannabinoids on a molecular and cellular level. For example, cannabinoid activation of CB1 receptors, which are found on both pyramidal cells and interneurons, reduces the quantity of neurotransmitter released during an action potential; consequently, increased extracellular cannabinoid levels reduce both excitatory (glutamatergic) and inhibitory (GABAergic) transmission [10]. Furthermore, cannabinoids have been shown to interact with astrocytes [11], mitochondria [12], glycine receptors [13], vanilloid receptors [14], potassium ion channels [15], and to reduce GABA and glutamate reuptake [16, 17]. Consequently, it is very difficult to extrapolate the emergent network level changes simply from a catalogue of effects cannabinoids have a cellular/molecular level.

Here, we studied the effects of Δ^9^-tetrahydrocannabinol (THC) on hippocampal networks during memory encoding using spiking activity recorded in rodents in-vivo performing the Delayed-NonMatch-to-Sample (DNMS) working memory task. Multivariate autoregressive (MVAR) models were used in both control and THC sessions to estimate feedforward and feedback dynamical filters, which are akin to the waveform shapes of the CA3→CA1 EPSP and CA1 afterhyperpolarization, respectively [18]. MVAR models, which are a type of linear nonparametric model, are ‘data-driven’ in the sense that they estimate model parameters directly from recorded neural spiketrains and, unlike more biologically realistic models, make very few *a priori* assumptions on the nature of the neural dynamics [19, 20]. This characteristic makes them particularly well suited for this study, since as previously mentioned the emergent effects of THC on neural circuits are highly complex and unclear. Overall our results suggest that cannabinoids impair memory encoding by functionally isolating CA1 from CA3 via reduced theta information flow and altered excitatory-inhibitory balance across the Schaffer collateral synapse.

## Results

### Changes in rate and temporal coding under cannabinoids

**Fig 1:**
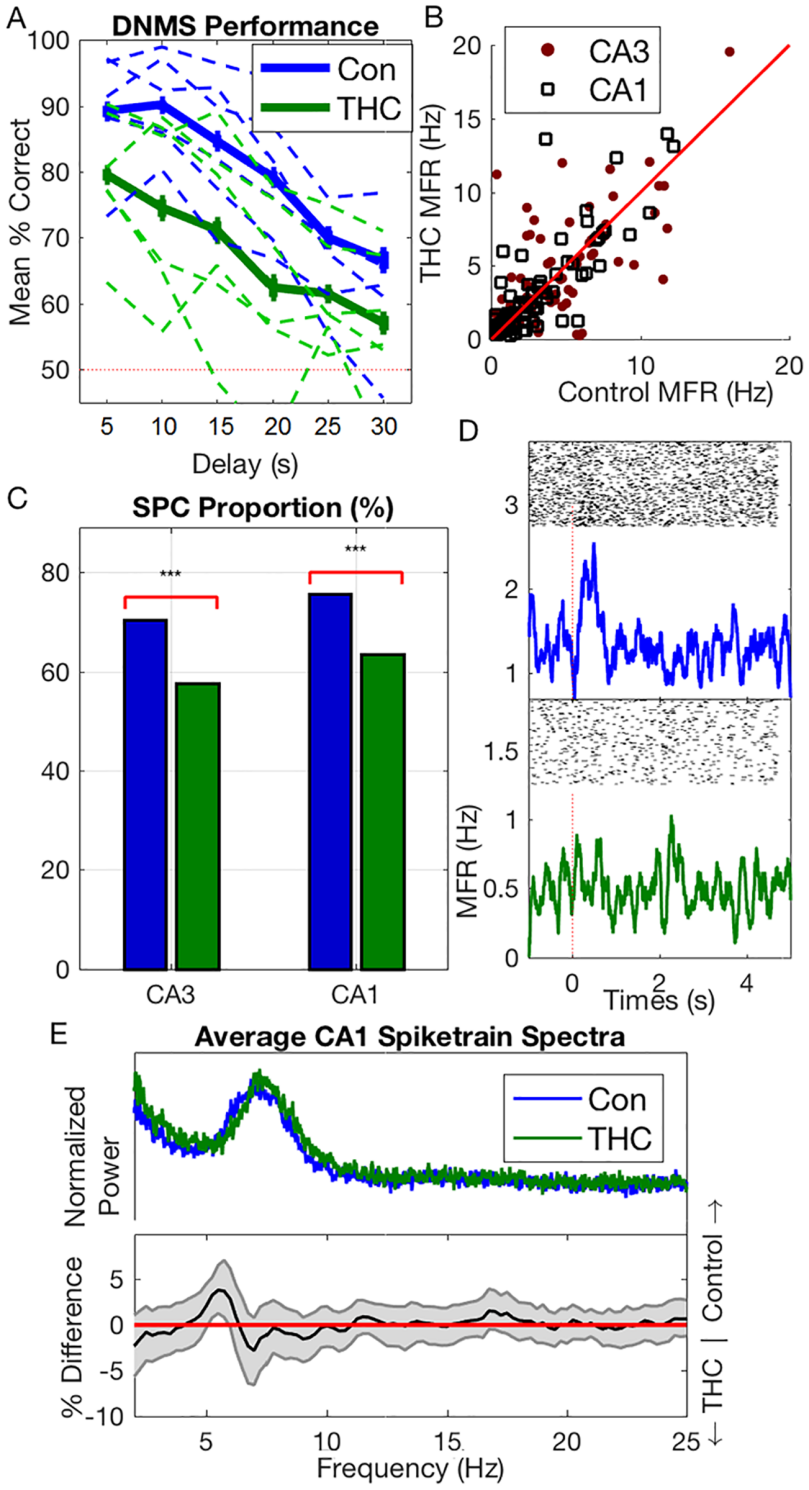
(A) Behavioral performance on Delayed-NonMatch-to-Sample (DNMS) task in both control and THC sessions. Dashed lines show individual animal performance, while solid lines show mean performance over all animals. Bars indicate SEM. Dashed red line indicates performance at chance level. (B) Individual neuron mean firing rate (MFR). (C) Sample-presentation cell proportion in CA3 and CA1 cells in control & THC sessions (*** = *P* < .001). (D) Example of a sample-presentation cell in a control session (top) which lost its firing specificity under THC (bottom). X-axis shows MFR (Hz) (E) Average CA1 spiketrain spectra (top). Bottom shows mean difference in *individual* cell spectra (thus it is not simply the difference between the signals in above which are averaged over whole population). Gray error bounds indicate 99% confidence bounds. In (B) and (E), only neurons recorded in at least one control & THC session were included. Results for neurons recorded in several control or THC sessions were averaged over those sessions.

To evaluate the effects of exogenous cannabinoids on the hippocampal network 1 mg/kg THC was injected intraperitoneally into *N* = 6 rodents during certain sessions while they were performing a DNMS task (S1 Fig). All data was previously used in a study on the effects of cannabinoids on hippocampal multifractality [21, 22]. Briefly, in the sample phase, the rats were presented one of two levers. After a variable length delay, both levers were presented in the match phase and the rat had to choose the opposite lever to receive a reward. On the behavioral level, it was found that THC reduced rodent-performance on the DNMS task by about 12.2 ± .6% (Fig 1a, [23]). This corresponds to a 24.4% impairment relative to baseline performance at 50%.

While performing the DNMS task, single-unit activity was recorded from the hippocampal CA3 and CA1 regions using a multi-electrode array. There were no significant mean firing rate (MFR) differences between THC sessions and control sessions in either CA3 or CA1 cells (P = .502, Fig 1b). No MFR differences were seen whether considering the entire session or only times around the DNMS sample phase, or whether considering all cells or only sample-presentation cells (see below). The lack of any cannabinoid-induced changes in firing rates at this dosage has been observed in previous studies [24, 25].

Two types of temporal coding were identified in the recorded spiketrains. First, on slower timescales, several neurons fired preferentially in response to lever presentation in the sample phase of the DNMS task [26]. It was found that THC reduced the proportion of sample-presentation cells in both CA3 and CA1 by roughly equal amounts (Δ = 13 ± 4%, *P* < .001; Fig 1c). Interestingly, some sample-presentation cells lost all of their preferential firing in THC sessions (Fig 1d); this contrasts with place cells whose receptive field stays largely intact under cannabinoids [27]. There was an insignificant trend connecting sample-presentation cell reduction with behavioral deficits (*R*^2^ = .27, *P* = .052, S3a Fig).

On faster timescales, it was found that several CA3 and CA1 neurons had theta band rhythmicity (4–7 Hz). Hippocampal theta oscillations are known to be intimately related to cognitive function [28–30] and have previously been linked to performance in the DNMS task [31]; furthermore, theta oscillations are known to be reduced by systemic injections of cannabinoids on both the single unit [24] and network level [32]. It was found that CA1 theta power was slightly but significantly reduced in THC sessions (Δ = 2.52%, *CI*: [.61, 4.4]%*P* = .004; Fig 1e). A similar, albeit slightly weaker, theta power reduction was seen in CA3 cells (Δ = 1.94%, *P* = .045; S2 Fig). Interestingly, in both cases, the significant reduction of theta power occurred at 5–6Hz, which is lower than the observed theta peak. Unlike previous results in a different task [24], the reduction in CA1 theta power was not found to be correlated with behavioral deficits in the DNMS task (*P* = .674, S3b Fig).

Overall, these results show that THC has minor effects on the actual neuronal spiketimes: quantity of spikes (MFR) was not affected and spike rhythmicity (theta oscillations) were only slightly affected. Furthermore behavioral deficits induced by cannabinoids could not be explained by any of these factors, which are the traditional markers of rate and temporal coding in the hippocampus.

### Systems analysis

The remainder of the study will focus on systems analysis of the Schaffer collateral synapse connecting CA3 to CA1, and how this synapse is affected by THC. Systems analysis aims to identify the input-output “blackbox” by which the input spiketrains are transformed into the output spiketrain. On a more abstract level, it aims to identify how the information encoded in CA3 is propagated into CA1. This is distinct from the *signal* analysis done in the previous section which only looks at features of individual spiketrains rather than the causal relationship between multiple spiketrains as done in systems analysis.

**Fig 2:**
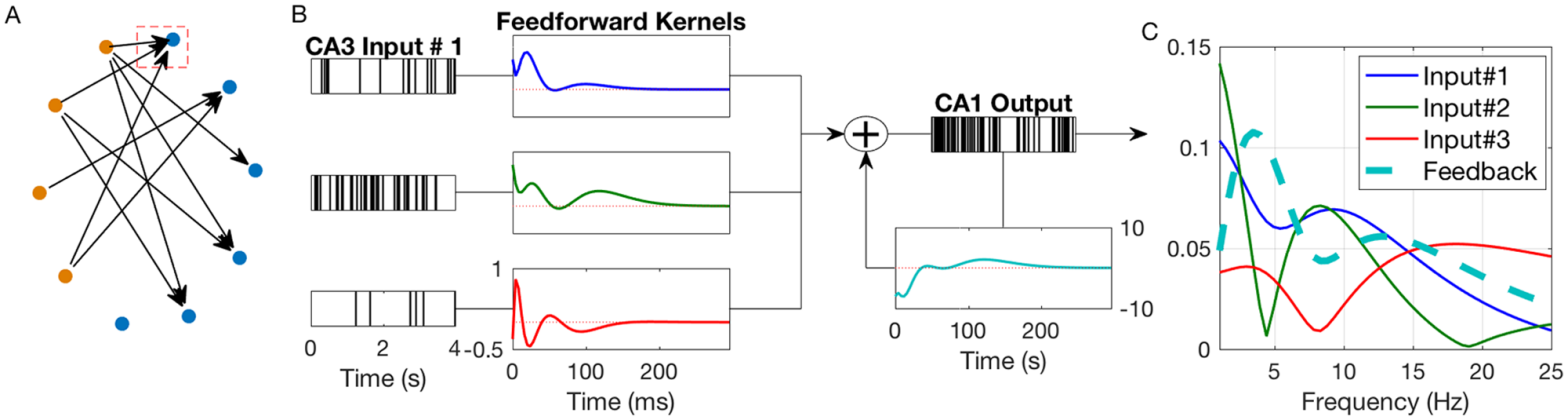
(A) Example connectivity grid of 4 CA3 neurons (orange) and 6 CA1 neurons (blue) recorded during a single session. Note that 1 CA1 neuron has no significant granger-causal inputs. Each line represents a causal connection between those neurons, as encapsulated by a feedforward filter. (B) Example system of CA1 neuron enclosed by the red box in (A). Diagram shows 3 input CA3 spiketrains followed by their respective feedforward filters which are summed with the feedback filter to generate the output CA1 spiketrain. All feedforward filter are plotted with the same y-axis scale. Dashed red line in filter boxes indicates x-axis. (C) Normalized filter spectra computed of feedforward and feedback filters from (B).

The relationship between an arbitrary number of input CA3 spiketrains and the output CA1 spiketrain was modeled using a multivariate autoregressive model described by Eq 1 and an example of which is pictured in Fig 2a. Each system consists of *N* input CA3 neurons and *N* feedforward filters describing the dynamical input-output relationship between the given CA3 and CA1 neurons (Fig 2b). Intuitively, these filters can be thought of as the EPSP elicited in the output CA1 neuron in response to an action potential (AP) in the input CA3 neuron. However, unlike EPSPs which traditionally only encapsulate ion-conductances from neurotransmitter-gated ion channels, the “blackbox” nature of the feedforward filters means they also include more complex dynamical effects such as dendritic integration, spike generation, active membrane conductances, and feedforward interneuronal inhibition (thereby allowing the filters between two pyramidal cells to be inhibitory). Each model also includes a feedback (autoregressive) filter which describes the effects of past output spikes onto the output present. This filter, which can be intuitively thought of as the afterhyperpotential (AHP) [33] includes intracellular processes such as the absolute and relative refractory periods, slow potassium conductances, and *I*_*h*_ conductances. It also includes more complex intercellular processes such as the recurrent connections between CA1 pyramidal cells and interneurons [34]. Neuronal connectivity was estimated using a stepwise input selection procedure. Filter parameters were estimated with Laguerre basis regression using neuronal activity around the sample phase. Model significance was verified using ROC plots and shuffling methods (see supplementary methods).

A representative connectivity grid from a recorded THC session with 10 recorded neurons (4 CA3, 6 CA1) is shown in Fig 2a. Fig 2b shows a sample system from this session between 3 CA3 pyramidal cells and 1 CA1 pyramidal cell. Note that two of the feedforward filters are excitatory (above the x-axis) while the third has both excitatory and inhibitory components, presumably arising through feedforward inhibition involving interneurons [35, 36]. The system also involves a feedback filter which shows a relatively long refractory period (∼40ms) followed oscillatory bursting activity. Oscillations in the CA1 pyramidal cell AHP are a well known phenomena caused by slow *K*^+^ and *I*_*h*_ conductances, and these oscillations are known to lead to theta resonances [18, 37, 38]. In order to study the filter oscillations more closely, the filter frequency spectra were plotted in Fig 2c. Both feedforward excitatory filters were found to have peaks in the high theta range (8–9 Hz). Intuitively, this can be understood to mean that information encoded in the theta range in these input neurons is preferentially transmitted to the output CA1 neuron. Furthermore, the feedback filter has a low theta resonance of 3.5 Hz. Significance metrics for the displayed system is shown in S4 Fig, and additional systems are shown in S5 Fig. All together 66% (707/1068) of all systems were found to be significant and 2139 feedforward and 707 feedback filters were obtained. THC was found to reduce the number of significant models per session (Δ = −7.4%, *P* = .011), but the predictive power of significant models, as measured by AUC (see supplementary methods), was unaltered (*P* = .24).

**Fig 3:**
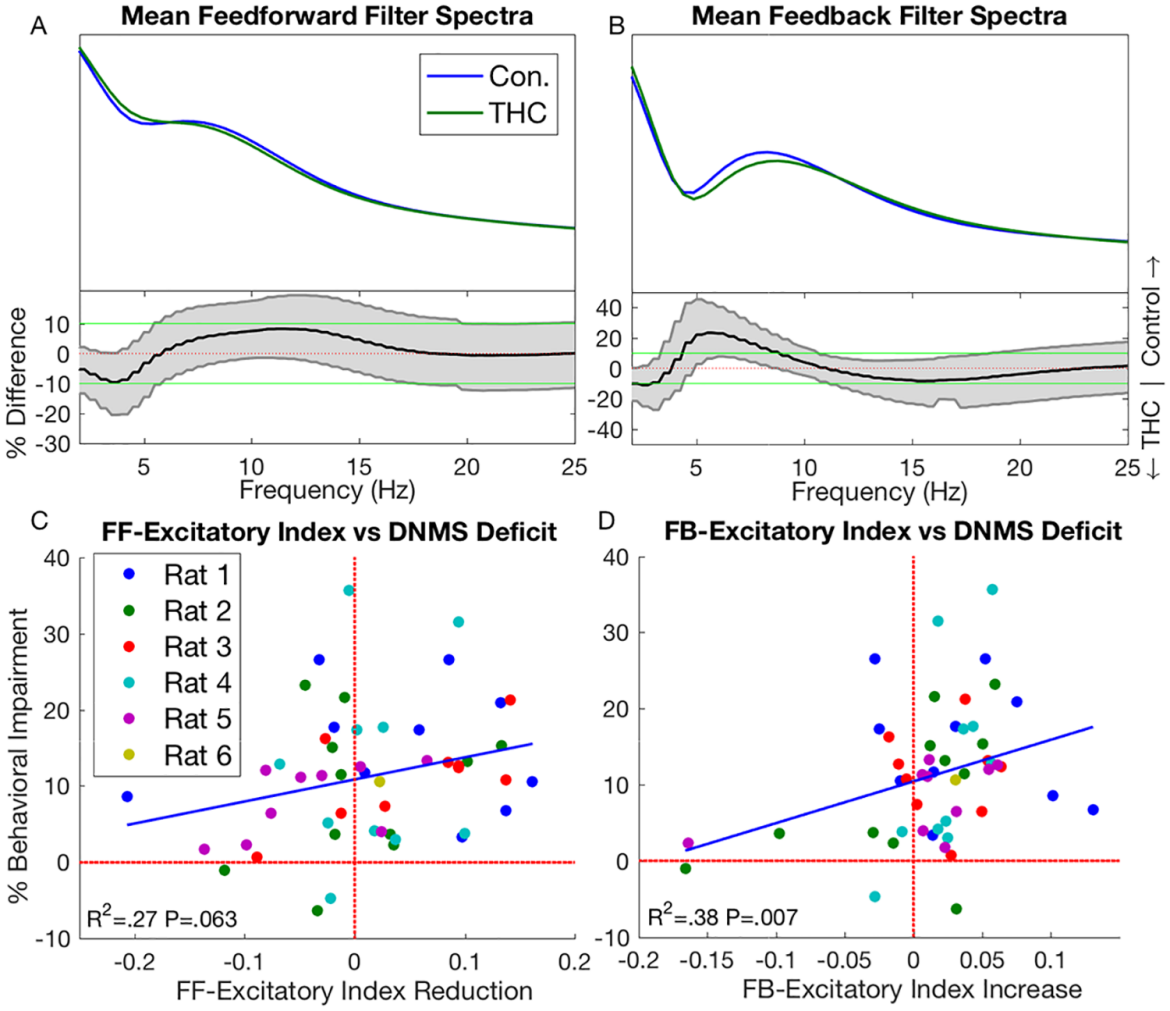
Average feedforward (A) and feedback (B) filter spectra in control and THC sessions (top), and their differences (bottom). Same format and analysis as Fig 1a. (C) Correlation between feedforward filter excitatory index (EI) reduction and behavioral deficits. Each point represents a specific THC session, with points of the same color coming from the same animal. X-axis shows reduction in feedforward EI, while y-axis shows reduction in behavioral performance. Both reductions were taken relative to control sessions (see supplemental methods). (D) Same as (C) but for feedback EI *increase*.

To study how THC affects system dynamics on a population level, we examined how features change in the entire sample of control and THC filters. The average filter frequency profile for both control and THC sessions is shown in Fig 3a and 3b (top). Both feedforward and feedback spectra are found to have clear theta band peaks, thus generalizing the trend seen in the example system of Fig 2. This is consistent previous reports which show that CA3 propagates strong theta rhythms to CA1 [39, 40] and also that CA1 is capable to generating endogenous theta rhythms [41]. THC produced a significant decline in the theta power of the feedback filters (Δ = 20.8%, *P* < .001; Fig 3b). Note that the feedback filter theta reduction is about 10x stronger than the theta reduction found in the CA1 spiketrain signals (Fig 1e). No reduction in theta power was found in the feedforward filters (*P* = .61, Fig 3a). This result suggests that cannabinoid-induced theta desynchronization results primarily from altered feedback properties rather than changes in CA3→CA1 dynamics.

Cannabinoids have been reported to affect network excitation-inhibition balance (EIB) [10, 42]. Particularly, there is much debate whether cannabinoids are pro- or anticonvulsants [4, 6, 8, 43, 44]. In order to examine the effects cannabinoids have on network EIB, we quantified the excitation of the estimated filters using a metric called the excitatory index (EI), which is the ratio between positive filter area and total filter area. It was found that THC had no significant effect on feedforward EI (*P* = .14); however, there was an insignificant trend showing that THC-induced decreases in feedforward EI were correlated with behavioral deficits (*R*^2^ = .27, *P* = .063, Fig 3c). Additionally, THC reduced the number of casually connected CA3-CA1 neuronal pairs (Δ = −8.9%, *P* < .001). These findings, together with the THC-induced decrease of CA3→CA1 significant models, suggest that THC reduces the causal influence CA3 neurons have on CA1 spiketimes. In other words, THC can be said to functionally isolate CA1 from CA3. It was also found that THC significantly increased feedback EI (Δ = 3.5%, *P* = .022) and that the increased feedback EI was correlated with behavioral deficits (*R*^2^ = .38, *P* = .007, Fig 3d).

### PDM analysis

The large quantity (>2800) and variability of the obtained filters describing the CA3→CA1 dynamic transformation presents a challenge of interpretation. Namely, how could one identify features from the entire filter population which are representative of the CA3→CA1 transformation rather than just the input-output relationship found in this or that particular pair of neurons. In essence this is an unsupervised learning problem which aims to identify hidden structure within the filter population for the purpose of knowledge discovery. Our group has developed the concept of the global principal dynamic modes (gPDMs) towards this effort [19, 45, 46]. The gPDMs are a system-specific and efficient basis set which contain the essential dynamic components of the filter population and are meant to be amenable to biological interpretation. One set of gPDMs were estimated from all (control and THC) obtained filters with the hypothesis that THC would primarily change the expression strength of the gPDMs rather than their specific shapes.

**Fig 4:**
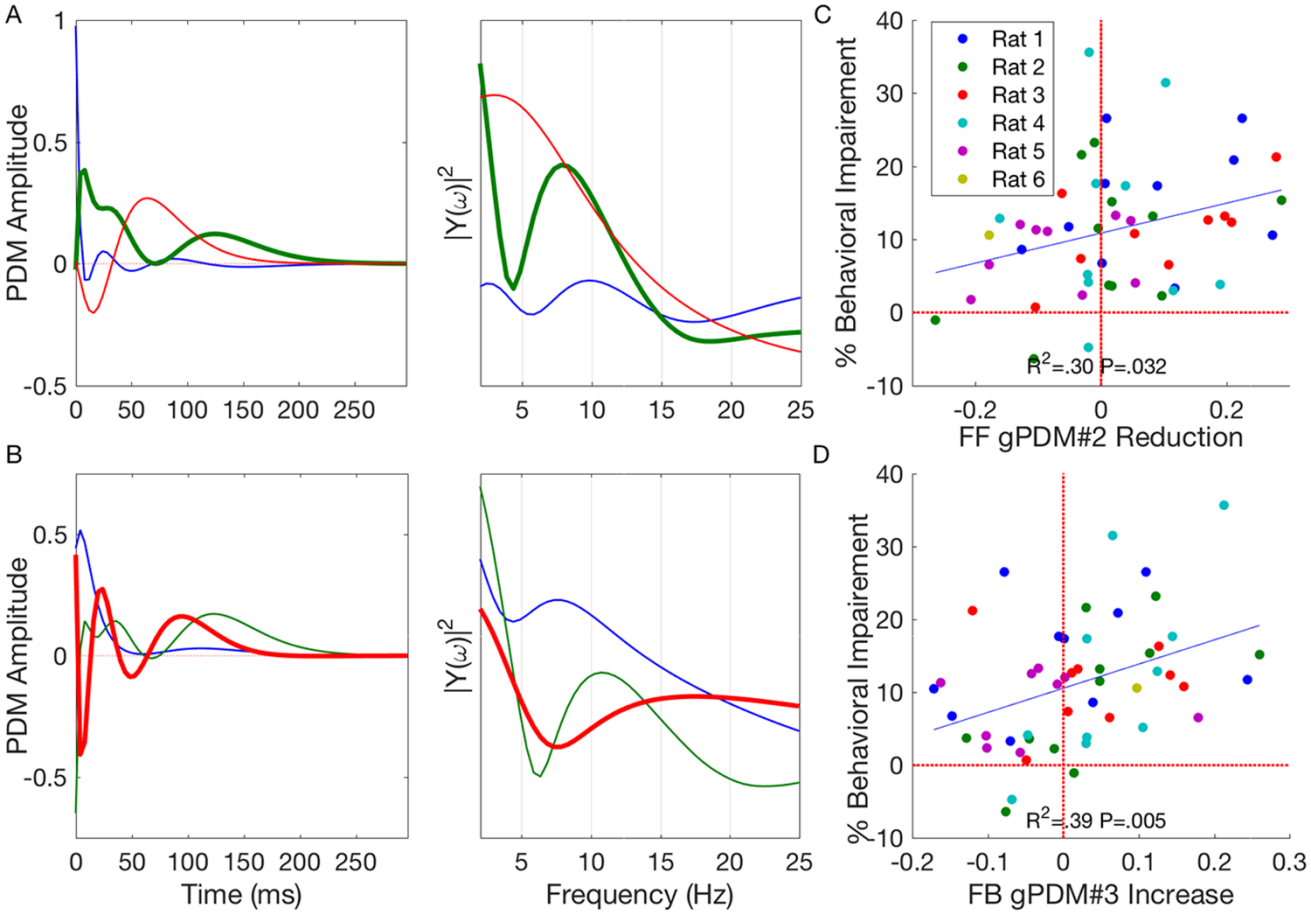
Feedforward (A) and feedback (B) global principal dynamic modes (gPDMs) in both the time (left) and frequency domain (right). Reductions in 2nd feedforward gPDM (C) and increases in 3rd feedback gPDM (D) were found to be correlated with behavioral deficits. Same format as Fig 3.

Fig 4a and 4b shows the obtained feedforward and feedback gPDMs in both the time and frequency domain. Once again, the feedforward and feedback gPDMs represent the dominant independent componenets of feedforward and feedback kernels, respectively. The first feedforward gPDM was found to have almost all its energy in the 1st time bin, with an immediate decline thereafter. This gPDM represents near concurrent firing between CA3 and CA1 neurons and presumably results from both direct CA3→CA1 connections via the Schaffer collateral synapse [47, 48] and common inputs from the entorhinal cortex [49, 50]. The third feedforward gPDM, which is characterized by an initial inhibitory phase, presumably represents feedforward interneuronal inhibition which is prevalent in the CA3→CA1 connection [35, 36]. THC was not found to influence the strength of either of these gPDMs (*P* = .76, *P* = .60; S6 Fig). The second feedforward gPDM which is characterized by sustained and oscillatory excitation was found to have a strong theta peak in the frequency domain. Furthermore, it was found that THC-induced declines in the strength of this gPDM were correlated with behavioral deficits (*R*^2^ = .30, *P* = .032; Fig 4c).

The three obtained feedback gPDMs are shown in Fig 4b. These gPDMs express the essential feedback dynamics found in CA1 neurons. As previously mentioned, these dynamics arise through the combination of intracellular processes such as the AHP and extracellular processes such as recurrent connections between CA1 pyramidal cells and interneurons. It was found that THC-induced increases in the third feedback gPDM were correlated with behavioral deficits (*R*^2^ = .39, *P* = .005; Fig 4d). This correlation was not seen in either of the first two feedback gPDMs (*P* = .32, *P* = .75; S6 Fig). Notably, the 3rd feedback gPDM was seen to be “theta-blocking” in the frequency domain due to its trough at 8 Hz. This gPDM counteracts the 1st “theta-promoting” feedback gPDM and disrupts theta oscillations in the CA1 neuron. The THC-induced changes in the feedforward and feedback theta gPDMs paint a more complete picture of the CA1 theta reductions seen in Fig 1e. Namely, they attribute the theta losses to specific feedforward and feedback dynamical filters which may potentially be traced to specific biophysical mechanisms. Furthermore, changes in these dynamical filters have been specifically correlated with behavioral deficits, which could not be done with theta reductions in the CA1 signal (S3 Fig).

## Discussion

The current study uses ‘data-driven’ nonparametric system dynamics modeling tools to study the effects of THC on the Schaffer Collateral synapse in rodents. The chief findings of the study can be summarized as: (1) THC induced little or no change in traditional rate and temporal coding metrics such as MFR and theta power, (2) THC altered the CA1 excitatory-inhibitory balance by reducing feedforward influence from CA3 while increasing feedback excitation from CA1, (3) THC reduced theta information flow through the Schaffer collateral synapse, and (4) The magnitudes of both of the previous effects were directly correlated with the severity of behavioral deficits induced by THC. Overall these results suggest the conclusion that THC impairs memory encoding by functionally isolating CA1 from CA3.

From a computational perspective, the nonparamteric modeling methods used in this study proved successful in studying the network level effects of cannabinoids since, unlike biophysical models, all model parameters where estimated directly from recorded data and very few *a priori* assumptions were made about the effects of THC [19, 20, 51]. The global principal dynamic modes (gPDMs), which were derived from MVAR filters of the entire population of neurons, further extracted hidden dynamical structure from ‘noisy’ neuron-neuron variability. Importantly, THC-induced changes in the gPDMs were directly correlated with behavioral impairments, thus justifying their utility. Furthermore, while most in-vivo studies on THC analyze macro level signals such as ECoG and EEG, this work adds to a relatively small body of literature which analyzes the effects of THC on neuronal population spiking activity. Finally, to our knowledge, this is the first work which examines the effect of THC on neuronal systems dynamics, or the causal interactions between signals, rather than on neuronal signals themselves.

It was found that THC increased feedback excitatory index in CA1 and that the magnitude of this effect was correlated with behavioral deficits. We hypothesize that this is due to reduced feedback inhibition from CA1 cholecystokinin (CCK)-containing cells. While CCK cells only make up only 13.9% of interneurons [52], they express significantly more CB1 receptors than any other cell in the hippocampus [53], and their primary output is to CA1 pyramidal cells [52]. Increased THC concentrations would reduce CCK interneuron output by (1) reducing the amount of GABA they release per action potential (2) reducing their MFR due to reduced glutamatergic input from principal cells in both CA3 and CA1 [54, 55].

It was also found that THC reduced the number of casually connected CA3-CA1 neuronal pairs; furthermore there was an interesting but insignificant trend for THC-induced deficits in feedforward excitation to lead to behavioral deficits. This trend may prove to be significant given a higher sample size. We hypothesize that this reduced feedforward influence is caused by decreased glutamate release from CA3 pyramidal cells due to CB1 receptor activation by THC [56]. Even though pyramidal cells have much lower densities of CB1 receptors than interneurons [53, 57], there is evidence that CB induced reduction of excitation is larger than these relative densities suggest. Principal cells outnumber interneurons 20:1 in CA1 [50] and their CB1 receptors were found to be several fold more efficacious than those of interneurons [58]. Further, lower baseline activation levels of CB1 receptors on principal cells than on interneurons suggest they would be disproportionately activated by CB agonists [59]. Altogether, the decreased feedback inhibition and feedforward excitation amount to a functional isolation, or breakdown in information flow between CA3 and CA1. We suggest that this functional isolation is responsible for the behavioral impairments seen in the DNMS task.

The ‘functional isolation’ hypothesis is further supported by previous work which showed that the behavioral impairments caused by cannabinoids in the DNMS task were similar to those seen with a full pharmacological lesion of the hippocampus [60] Given the centrality of CA3→CA1 information flow to hippocampal function, a functional isolation of these areas could indeed presumably lead to impairments similar to that of a full lesion. Relatedly, Goonawardena et al., 2010 [25] injected THC intraperitoneally at low 1 mg/kg doses as in this study and in higher doses of 3 mg/kg. They found that while both doses disrupted hippocampal synchrony, only the higher dose resulted in a reduction in pyramidal cell MFR. This suggests that at the lower dose both previously described phenomena are at a net balance, while at the higher dose, the decrease in feedforward excitation overpowers the increase in feedback excitation and results in lower MFR. Finally, the hypothesis predicts a breakdown in the normal spiketime coordination between pyramidal cells and interneurons in CA1 circuits. The breakdown of this coordination, which has been extensively implicated in hippocampal oscillations [61, 62], could be responsible for the observed decrease in theta oscillations and information flow.

Although the current results only suggest this hypothesis, several experiments could be done to further substantiate it. Feedforward and feedback kernels and gPDMs could be estimated at different doses of THC; the hypothesis would predict that different doses would effect the two processes independently, with one of the two processes potentially being more dominant at different THC levels. Significant developments in in-vivo synaptic patch clamping [63] and calcium imaging in recent years could be used to directly measure the drive of CCK cells and CA3 pyramidal cells onto CA1 pyramidal cells under THC.

Much research has been done investigating the effects THC and other cannabinoids have on seizures and epilepsy. Results so far have been mixed, with various studies showing that THC is both pro- and anticonvulsant [3–8]. The results from this study and the presented hypothesis suggest that THC inherently is not pro- or anti-convulsant but that its effects will depend on the dosage and the unique circuitry of every epileptic focus. Interestingly, a study by Rudenko et al., 2012 [6] has shown that indeed the effects of a CB1 agonist were dose dependant, with *lower* doses being anticonvulsant and higher doses being proconvulsant. Finally, this study suggests that in order to truly understand the effects of THC on epileptic circuits, one must study the systems level changes in circuit dynamics rather than taking a reductionist approach and studying the effects of THC on any particular receptor or cell type.

The present study analyzed the effects of THC from both a signals and systems perspective—and found that systems analysis yielded much richer results. For example, while analysis of CA1 spiketrain signals showed a slight (2%) reduction in theta frequency, analysis of system kernels showed that the theta loss was primarily due to CA1 feedback dynamics whose kernels lost over 20% of their theta power, while theta power in feedforward kernels was unaffected. Furthermore, only systems analysis allows one to analyze predictive power, feedforward and feedback excitation, and EPSP and AHP waveform shape. Notably, the finding that feedforward influence decreased while feedback excitation increased could not have been observed using only signal analysis which would have only detected a constant MFR.

The present study also employed gPDMs as a means to extract the most significant information from the kernel dynamics estimated from several animals over several sessions [18, 19, 48, 64]. The utility of the gPDM method was justified by the finding that reductions in theta related gPDMs in a given session were directly correlated with behavioral deficits, showing that the gPDMs can isolate the particular dynamics which are most affected by THC. Furthermore, THC-induced theta power losses in spiketrain signals were not found to be correlated with behavioral deficits. Although in the present study, kernels and gPDMs were restricted to being linear in order to more easily quantify their overall strength and excitation (via the EI), future work will aim to identify the effects of THC on hippocampal nonlinear dynamics [51, 65].

## Methods

### Experimental procedures

N = 6 Male Long-Evans rats were trained to criterion on a two lever, spatial Delayed NonMatch-to-Sample (DNMS) task (see S1 Fig). Briefly, during the sample phase the rat was presented one of two levers (left or right). After a delay phase ranging from 1–30 seconds, the rat was presented both levers and had to choose the opposite level in order to attain a reward. Each rodent underwent 16–25 sessions of the task, which were roughly evenly divided between control and THC sessions, wherein the rodent was intraperitoneally administered 1 mg/kg of body weight Δ^9^-tetrahydrocannabinol (THC), an exogenous cannabinoid found in marijuana. During the task, spike trains were recorded in-vivo with multi-electrode arrays implanted in the left and right CA3 and CA1 regions of the hippocampus. In an effort to acquire a consistent cognitive state, only spiking activity around the sample phase of the task was used. Spikes from multiple trials were sorted, time-stamped, and concatenated into a discretized binary time series using a 4ms bin. For more details on the experimental setup, see supplementary methods.

### Model configuration and estimation

**Fig 5:**
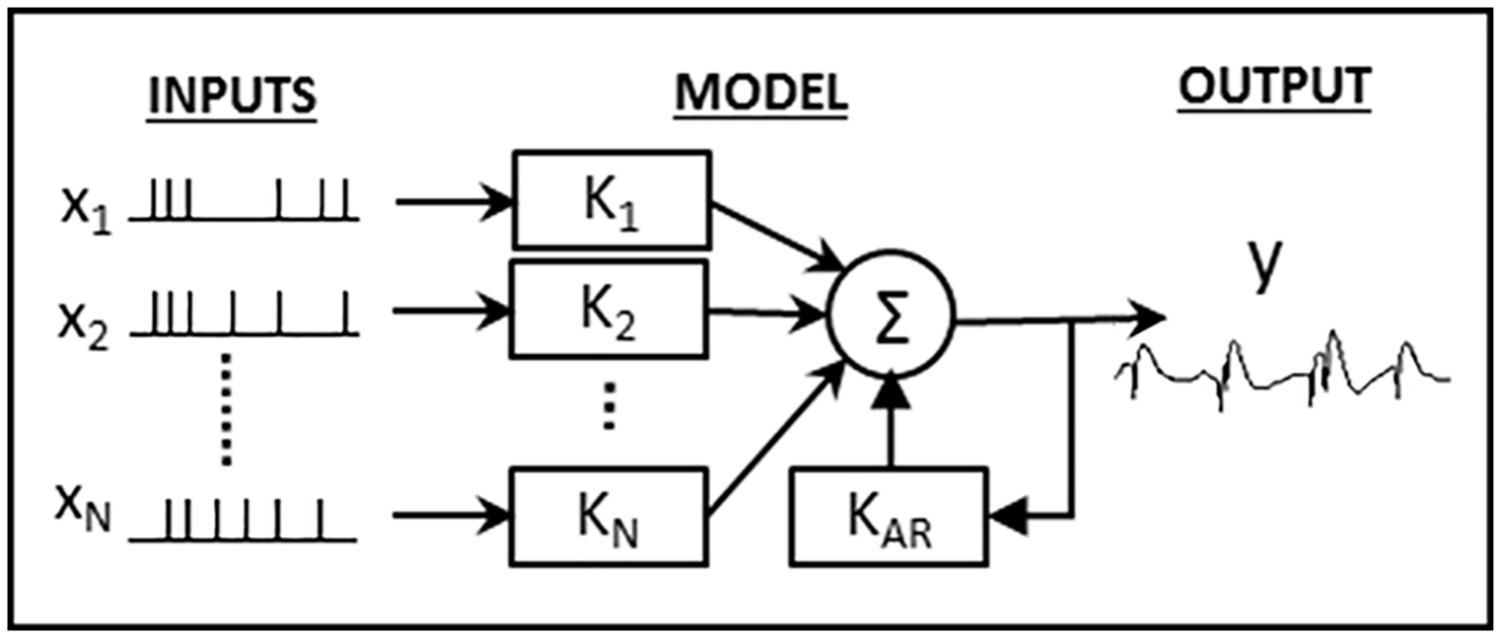
Model configuration. Each model has *N* point-process inputs which each go through a linear filter, *K*_*i*_. These inputs are then summed with the output of the feedback filter, *K*_*AR*_ to generate the final output, *y*(*t*), which is a continuous signal.

Nonparametric multiple-input linear autoregressive models were used to model the dynamical transformation between input and output spike trains (see Figs 2 and 5) [18, 51]. Each model consisted of a feedforward component, reflecting the effect of the *N* input cells on the output cell and a feedback (autoregressive) component reflecting the subthreshold and suprathreshold effects the output cell has on itself. Thus, the output *y*(*t*) is calculated as:

$$\begin{array}{c} y\left(t\right)=\sum_{n=1}^{N}\sum_{\tau =0}^{M}\;k_{n}\left(\tau \right)x_{n}\left(t-\tau \right)+\sum_{\tau =1}^{M+1}\;k_{AR}\left(\tau \right)y\left(t-\tau \right) \end{array}$$ (1)

where *k*_*n*_ reflects the feedforward filter of input *x*_*n*_(*t*), and *k*_*AR*_ reflects the feedback filter. In order to reduce the number of model parameters and thereby increase parameter stability, we applied the Laguerre expansion technique to expand the feedforward and feedback filters over *L* Laguerre basis functions (see supplementary methods).

Effective connectivity between neurons was assessed using a Granger causality-like approach. For each output CA1 neuron, input CA3 neurons were selected in a forward stepwise procedure whereby only neurons which help predict the output CA1 spike activity were included in the model. After all input neurons were selected, a Monte Carlo approach was used to assess model significance. A model was deemed significant if the CA3 inputs could predict the output CA1 activity significantly better (*P* < .0001) than randomly permuted versions of the inputs. See supplementary methods for more details.

### Principal dynamic modes

The global principal dynamic modes (gPDMs) were obtained in a two step process: first, all filters of each input from every animal were concatenated in a rectangular matrix. Then singular value decomposition (SVD) was performed on the rectangular matrix to obtain all the significant singular vectors, which are the gPDMs. It was found that 3 gPDMs were sufficient to describe the linear dynamics both the population of feedforward and feedback filters. gPDM strength in a given filter was computed by taking the dot product between the gPDM and the filter. gPDM strength in a given session was computed by taking the average gPDM strength in every filter of that session.

## Supporting information

S1 Appendix Supplementary methods. (PDF)

S1 Fig Schematic of the DNMS task. First the rat is presented with one of two levers (sample presentation), which it presses (sample response). Then following a delay phase, the rat is presented with both levers (Nonmatch), of which it must press the opposite level from which it was presented in order to successfully complete the task.
(TIF)

S2 Fig CA3 spectra mean frequency and differences. Same format as Fig 1e. A weak but significant trend was found for declining CA3 theta oscillations (Δ = 1.94%, *P* = .045).
(EPS)

S3 Fig **(A) A suggestive but insignificant relationship was found between the THC-induced decrease in the mean number of sample-presentation cells and behavioral performance** (*R*^2^ = .265, *P* = .052). (B) No relationship was found between reductions in CA1 theta power and behavioral impairment (*P* = .67). Format is same as Fig 3.
(EPS)

S4 Fig **(A) ROC plot (see supplementary methods) for model shown in** Fig 2 **showing model predictive power**. The light blue line (TPR = FPR) indicates a model with no predictive power. (B, C) Examples of Monte Carlo simulations: For each model, 40 surrogate models with shuffled inputs were generated. The Fisher z-scores of these models, which are derived from *ρ*, were plotted as a histogram, while the true *ρ* value is the plotted dashed red line. The P value for the hypothesis that the true *ρ* value is greater than the simulated *ρ* values is printed above the graphs. Models were deemed significant if *P* < .0001. (B) shows the results for the model in Fig 2, which was deemed significant. (C) shows an insignificant model.
(EPS)

S5 Fig 4 additional systems are presented. Left column shows all system filters, including feedback filter (dashed line) in the time domain. Middle column shows the filters in the frequency domain and right column shows the ROC plots of the models. All these models were found to have significant predictive power in Monte Carlo tests.
(EPS)

S6 Fig **Top Row: neither the first (middle column) nor third feedforward gPDM were found to be significantly correlated with THC induced behavioral deficits**. Bottom Row: neither the first (middle column) nor second feedback gPDM were found to be significantly correlated with THC induced behavioral deficits. Format is same as Fig 3.
(EPS)
