# Supplementary material for: Cannabinoids disrupt memory encoding by functionally isolating hippocampal CA1 from CA3
Source: PLoS Comput Biol. 2017 Jul 7;13(7):e1005624. doi: 10.1371/journal.pcbi.1005624 (PMC5521875; doi:10.1371/journal.pcbi.1005624)
Supplement: S1 Appendix — (PDF) [file pcbi.1005624.s001.pdf]

Supplementary Methods

All data was previously used in a study on the effects of cannabinoids on hippocampal multifractality [1, 2])

Animals

Subjects were Long–Evans rats (Harlan) aged 4–6 months (n = 6) individually housed and allowed free access to food with water regulation to maintain 85% of ad libitum body weight during testing. All animal protocols were approved by the Wake Forest University Institutional Animal Care and Use Committee, in accordance with the Association for Assessment and Accreditation of Laboratory Animal Care and the National Institute of Health Guide for the Care and Use of Laboratory Animals (NIH Publication No. 8023).

Apparatus

The behavioral testing apparatus for the delayed nonmatch-to-sample (DNMS) task is the same as reported in other studies [3] and consisted of a 43x43x50 cm Plexiglas chamber with two retractable levers (left and right) positioned on either side of a water trough on the front panel. A nosepoke device (photocell) was mounted in the center of the wall opposite the levers with a cue light positioned immediately above the nosepoke device. A video camera was mounted on the ceiling and the entire chamber was housed inside a commercially built sound-attenuated cubicle.

DNMS Task

The DNMS task consisted of three main phases: Sample, Delay and Nonmatch. The sample phase initiated the trial when either the left or right lever was extended (50% probability), requiring the animal to press it as the Sample Response (SR). The lever was then retracted and the Delay phase of the task initiated, as signaled by the illumination of a cue light over the nosepoke photocell device on the wall on the opposite side of the chamber. At least one nosepoke (NP) was required following the delay interval which varied randomly in duration (1-30 s) on each trial during the session. The Nonmatch phase began when the delay timed out, the photocell cue light turned off and both the left and right levers on the front panel were extended. Correct responses consisted of pressing the lever in the Nonmatch phase located in the spatial position opposite the SR (nonmatch response: NR). This produced a drop of water (0.4 ml) reward in the trough between the two levers. After the NR the levers were retracted for a 10.0 second intertrial interval (ITI) before the next Sample lever was presented to begin the next trial. A lever press at the same position as the SR (match response) constituted an “error” with no water delivery and turned off of the chamber house lights for 5.0s and the next trial was presented 5.0 s later. Individual performance was assessed as % NRs (correct responses) with respect to the total number of trials (80-100) per daily (1 hr) sessions.

Drug Preparation & Administration

Δ^9-tetrahydrocannabinol (THC) was obtained from the National Institute on Drug Abuse as a 50 mg/ml solution in ethanol. Detergent vehicle was prepared from Pluronic F68 (Sigma, St. Louis, MO), 20 mg/ml in ethanol. THC was added to the detergent-ethanol solution (0.5 ml of either THC), and then 2.0 ml of saline (0.9%) was slowly added to the ethanol-drug solution. The solution was stirred rapidly and placed under a steady stream of nitrogen gas to evaporate the ethanol (~10 min). This resulted in a detergent-drug suspension (12.5 mg/ml THC), which was sonicated and then diluted with saline to final injection concentrations (0.5-2.0 mg/ml THC). On drug administration days, animals were injected intraperitoneally with the drug-detergent solution (1 mg/kg) ~10 min before the start of the behavioral session. Our experience with these experiments has shown that performance after vehicle injection is not significantly different than no injection, and therefore was omitted during this series of experiments to minimize risk of infection to the animals. At least two no injection days were imposed between each drug-testing session. All drug solutions were mixed fresh each day.

Surgery

All surgical procedures conformed to National Institutes of Health and Association for Assessment and Accreditation of Laboratory Animal Care guidelines, and were performed in a rodent surgical facility approved by the Wake Forest University Institutional Animal Care and Use Committee. After being trained to criterion performance level in the DNMS task animals were anesthetized with ketamine (100 mg/kg) and xylazine (10 mg/kg) and placed in a stereotaxic frame. Craniotomies (5mm-diameter) were performed bilaterally over the dorsal hippocampus to provide for implantation of 2 identical array electrodes (Neurolinc, New York, NY), each consisting of two rows of 8 stainless steel wires (diameter: 20 μm) positioned such that the geometric center of each electrode array was centered at co-ordinates 3.4 mm posterior to Bregma and 3.0 mm lateral (right or left) to midline [4]. The array was designed such that the distance between two adjacent electrodes within a row was 200 μm and between rows was 400 μm to conform to the locations of the respective CA3 and CA1 cell layers. The longitudinal axis of the array of electrodes was angled 30° to the midline during implantation to conform to the orientation of the longitudinal axis of the hippocampus, with posterior electrode sites more lateral than anterior sites. The electrode array was lowered in 25-100 μm steps to a depth of 3.0 - 4.0 mm from the cortical surface for the longer electrodes positioned in the CA3 cell layer, leaving the shorter CA1 electrodes 1.2 mm higher with tips in the CA1 layer. Extracellular neuronal spike activity was monitored from all electrodes during surgery to maximize placement in the appropriate hippocampal cell layers. After placement of the array the cranium was sealed with bone wax and dental cement and the animals treated with buprenorphine (0.01–0.05 mg/kg) for pain relief over the next 4-6 hrs. The scalp wound was treated periodically with Neosporin antibiotic and systemic injections of penicillin G (300,000 U, intramuscular) were

given to prevent infection. Animals were allowed to recover from surgery for at least 1 week before continuing behavioral testing [5].

Electrophysiological Monitoring & Preprocessing

Animals were connected by cable to the recording apparatus via a 32-channel headstage and harness attached to a 40-channel slip-ring commutator (Crist Instruments, Hagerstown, MD) to allow free movement in the behavioral testing chamber. Single neuron action potentials (spikes) were isolated by time-amplitude window discrimination and computer-identified individual waveform characteristics using a multi-neuron acquisition (MAP) processor (Plexon Inc., Dallas, TX, USA). Single neuron spikes were recorded daily and identified using waveform and firing characteristics within the task (perievent histograms) for each of the DNMS events (SR, LNP & NR). To maintain waveform shape across days, all recorded data was concatenated into one file (separately for each rat) and offline sorting was performed using principal component analysis, peak-valley, and nonlinear energy algorithms in Offline Sorter (Plexon Inc., Dallas, TX, USA). Hippocampal neuron ensembles used to distinguish recording phases and drug treatment conditions consisted of 10-30 single neurons, each recorded from a separate identified electrode location on either of the bilateral arrays. All isolated spike trains contained no less than a 1 ms gap at the center of the autocorrelogram. No effort was made to differentiate between principal cells and interneurons. Previous work has shown that hippocampal neurons recorded with the same waveform from the same electrodes exhibit consistent mean, baseline and DNMS task modulated firing rate alterations [6, 7], and therefore individual neurons were treated as the same when recorded over multiple days. A total of 189 neurons recorded during 5,143 recording phases were analyzed in the reported experiments.

Sample-Response Cell Identification

Prior studies from this laboratory have identified hippocampal neurons recorded as above by “Functional Cell Types” (FCTs) described by different behavioral correlates of DNMS task-related events such as lever position and/or phase of the task [7, 8]. Sample-response cells, a subtype of FCTs, were identified by first constructing a smoothed (51 bin) perievent histogram around the sample presentation phase of the DNMS task. The neurons background firing rate mean and variance were calculated from activity 3.5-5s after sample presentation. If the neuron’s MFR from the 2 second window around sample presentation was 4 standard deviations greater than its MFR from the background period it was classified as a sample-response cell. It should be noted that for the purpose of this paper other FCTs such as those which respond to a specific lever (left/right) or trial-type cells were not considered [9].

Laguerre Expansion Technique

In order to apply the Laguerre expansion technique [10], the input and output data records were first convolved with the Laguerre functions:

$$v_{x_i}^{(l)} = \sum_{\tau=0}^{M} b_l(\tau) x_i(t-\tau) \tag{1}$$

$$v_{y}^{(l)} = \sum_{\tau=0}^{M} b_l(\tau) y(t-\tau) \tag{2}$$

where b_l is the lth Laguerre basis function. By first convolving with the Laguerre basis functions, the dynamical effects of the past input epochs are removed and we are left with a simple regression of contemporaneous data. Substituting the above equations into equation 1 (main text), we have:

$$y(t) = k_0 + \sum_{n=1}^{N} \sum_{l=1}^{L} c_{l,x_i}(l) v_{l,x_i}(t) + \sum_{l=1}^{L} c_{l,y}(l) v_{l,y}(t) \tag{3}$$

where c_{l,x_i} and $c_{l,y}$ are the feedforward and feedback Laguerre expansion coefficients. To estimate model parameters, eq. 3 was cast in matrix form:

$$\boldsymbol{y} = \boldsymbol{V}\boldsymbol{c} + \epsilon \tag{4}$$

where $\boldsymbol{y}$ is the vector of all N output samples, $\boldsymbol{V}$ is the design matrix consisting of the convolved inputs, $\boldsymbol{c}$ are the model parameters to be estimated, and ϵ is the modeling error. Eq. 4 was solved using least squares regression (LSR). The memory of our system was fixed at 300ms, in accordance with previous studies [11,12]. The Laguerre parameter α was fixed at 0.6 to reflect this system memory [10].

Model Selection

In theory, the most predictive model would include all recorded inputs. However, such a model would be susceptible to overfitting, and would not reveal which neurons are causally connected to each other. To overcome this issue a forward step-wise selection procedure was used to minimize overfitting and prune out all inputs which are not causally related to the output [13]. Given an output cell and M potential input cells recorded during the same session, the following steps were used to select the N input cells which are causally connected to the output cell. First, the data was divided into training (in-sample) and testing (out-of-sample) sets. Then, M single-input single-output (SISO) models were constructed with each of the potential inputs. The model whose predicted output had the highest correlation, as measured by the Pearson correlation-coefficient, ρ, with the actual output was selected. Afterwards, N-1 models were constructed with two inputs: the previously selected input and one of the remaining potential inputs. If any of the inputs were able to raise ρ, the input which raised ρ the

most was selected; otherwise, the procedure was ended, and only 1 input was selected. This procedure was repeated until either none of the inputs were able to raise ρ, or all M potential neurons were selected. The N selected neurons were then used as the model input.

Model Validation

To avoid overfitting, Monte Carlo style simulations were used to select those models which represent significant causal connections between input and output neurons and do not just fit noise [14]. The following procedure was used: in each run the real input was randomly permuted with respect to the output. A model was then generated between the permuted input and the real output, and the Pearson correlation coefficient, ρ_i, was obtained as a metric of performance. T=40 such simulations were conducted for each output and a set of performance metrics, $\{\rho_i\}_i^T$, was obtained. Then, using Fisher's transformation, we tested the hypothesis, H_0, that ρ was within the population of $\{\rho_i\}$. If this hypothesis could be rejected at the 99.99% significance level, the model was deemed significant. The very conservative threshold ($P < .0001$) was used due to the large number of comparisons being made.

Statistical Analysis

Unless otherwise noted, the unpaired Mann-Whitney U test was used to access whether significant differences exist between two samples. This test was used since it does not assume a normal distribution, and much of our data was found to be skewed/nonnormal. Shift estimates (Hodges-Lehman) and confidence intervals were estimated as prescribed by Higgins, 2003 [15]. In order to estimate the scale estimate, or the ratio between two samples, the data was first log-transformed and then scale estimate was taken to be the antilog of the shift estimate. The χ^2 test was used to compare proportions.

In addition to the Pearson correlation coefficient, ρ, Receiver Operating Characteristic (ROC) curves were used to visualize model performance. ROC curves plot the true positive rate against the false positive rate over the putative range of threshold values for the continuous output, y [14]. The area under the curve (AUC) of ROC plots are used as a performance metric of the model, and have been shown to be equivalent to the Mann-Whitney two sample statistic [16]. The AUC ranges from 0 to 1, with 0.5 indicating a random predictor and higher values indicating better model performance. The ρ and AUC metrics were chosen as they measure the similarity between a continuous 'prethreshold' signal and a spike train. The continuous 'prethreshold' signal was chosen over adding a threshold trigger and comparing true output spike train with an output 'post-threshold' spike train for two reasons. First, this allows us to avoid specifying the threshold trigger value, which relies on the somewhat arbitrary tradeoff between true-positive and false-negative spikes [17]. Also, similarity metrics between two spike trains often require the specification of a 'binning parameter' to determine the temporal resolution of the metric [18,19].

References

1. Fetterhoff D, Opris I, Simpson SL, Deadwyler SA, Hampson RE, Kraft RA. Multifractal analysis of information processing in hippocampal neural ensembles during working memory under Δ 9-tetrahydrocannabinol administration. Journal of Neuroscience Methods. 2014;.

2. Fetterhoff D, Kraft RA, Sandler RA, Opris I, Sexton CA, Marmarelis VZ, et al. Distinguishing cognitive state with multifractal complexity of hippocampal interspike interval sequences. Frontiers in Systems Neuroscience. 2015;9.

3. Hampson RE, Deadwyler SA. Cannabinoids reveal the necessity of hippocampal neural encoding for short-term memory in rats. The Journal of Neuroscience. 2000;20(23):8932–8942.

4. Paxinos G, Watson C. The rat brain in stereotaxic coordinates 2nd edn. Academic Press, New York. 1986;11(4):237–243.

5. Berger TW, Song D, Chan RH, Marmarelis VZ, LaCoss J, Wills J, et al. A hippocampal cognitive prosthesis: multi-input, multi-output nonlinear modeling and VLSI implementation. Neural Systems and Rehabilitation Engineering, IEEE Transactions on. 2012;20(2):198–211.

6. Deadwyler SA, Bunn T, Hampson RE. Hippocampal ensemble activity during spatial delayed-nonmatch-to-sample performance in rats. Journal of Neuroscience. 1996;16(1):354–372.

7. Hampson RE, Simeral JD, Deadwyler SA. Distribution of spatial and nonspatial information in dorsal hippocampus. Nature. 1999;402(6762):610–614.

8. Goonawardena AV, Robinson L, Hampson RE, Riedel G. Cannabinoid and cholinergic systems interact during performance of a short-term memory task in the rat. Learning & Memory. 2010;17(10):502–511.

9. Hampson RE, Song D, Chan RH, Sweatt AJ, Riley MR, Goonawardena AV, et al. Closing the loop for memory prosthesis: Detecting the role of hippocampal neural ensembles using nonlinear models. Neural Systems and Rehabilitation Engineering, IEEE Transactions on. 2012;20(4):510–525.

10. Marmarelis VZ. Nonlinear dynamic modeling of physiological systems. Wiley-Interscience; 2004.

11. Song D, Chan RH, Marmarelis VZ, Hampson RE, Deadwyler SA, Berger TW. Nonlinear dynamic modeling of spike train transformations for hippocampal-cortical prostheses. Biomedical Engineering, IEEE Transactions on. 2007;54(6):1053–1066.

12. Lu U, Song D, Berger TW. Nonlinear dynamic modeling of synaptically driven single hippocampal neuron intracellular activity. Biomedical Engineering, IEEE Transactions on. 2011;58(5):1303–1313.

13. Song D, Chan RH, Marmarelis VZ, Hampson RE, Deadwyler SA, Berger TW. Nonlinear modeling of neural population dynamics for hippocampal prostheses. Neural Networks. 2009;22(9):1340–1351.

14. Zanos TP, Courellis SH, Berger TW, Hampson RE, Deadwyler SA, Marmarelis VZ. Nonlinear modeling of causal interrelationships in neuronal ensembles. Neural Systems and Rehabilitation Engineering, IEEE Transactions on. 2008;16(4):336–352.

15. Higgins JJ. Intoruction to Modern Nonparametric Statistics; 2003.

16. Hanley JA, McNeil BJ. The meaning and use of the area under a receiver operating characteristic (ROC) curve. Radiology. 1982;143(1):29–36.

17. Marmarelis VZ, Shin DC, Song D, Hampson RE, Deadwyler SA, Berger TW. Nonlinear modeling of dynamic interactions within neuronal ensembles using Principal Dynamic Modes. Journal of computational neuroscience. 2013;34(1):73–87.

18. van Rossum MC. A novel spike distance. Neural Computation. 2001;13(4):751–763.

19. Victor JD, Purpura KP. Metric-space analysis of spike trains: theory, algorithms and application. Network: computation in neural systems. 1997;8(2):127–164.
