# Supplementary figures and images for: Cannabinoids disrupt memory encoding by functionally isolating hippocampal CA1 from CA3
Source: PLoS Comput Biol. 2017 Jul 7;13(7):e1005624. doi: 10.1371/journal.pcbi.1005624 (PMC5521875; doi:10.1371/journal.pcbi.1005624)

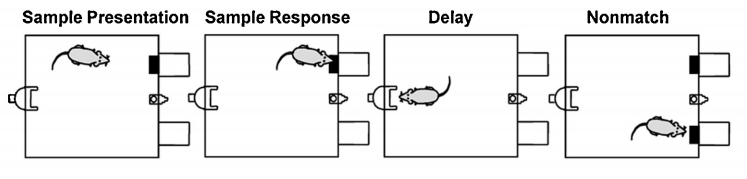

Supplement: S1 Fig — First the rat is presented with one of two levers (sample presentation), which it presses (sample response). Then following a delay phase, the rat is presented with both levers (Nonmatch), of which it must press the opposite level from which it was presented in order to successfully complete the task. (TIF) [file pcbi.1005624.s002.tif]
